# Predicting risk of chronic liver disease in Chinese adults: external validation of the CLivD score

**DOI:** 10.1016/j.jhep.2023.12.022

**Published:** 2024-01-04

**Authors:** Yuanjie Pang, Fredrik Åberg, Zhengming Chen, Liming Li, Christiana Kartsonaki, on behalf of the China Kadoorie Biobank Collaborative Group

**Affiliations:** 1 Department of Epidemiology & Biostatistics, School of Public Health, Peking University, Beijing 100191, China; 2 Key Laboratory of Epidemiology of Major Diseases ( Peking University), Ministry of Education; 3 Transplantation and Liver Surgery, HUCH Meilahti Hospital, Helsinki University Hospital and University of Helsinki, Helsinki, Finland; 4 Clinical Trial Service Unit & Epidemiological Studies Unit (CTSU), Nuffield Department of Population Health, University of Oxford, United Kingdom; 5 Peking University Center for Public Health and Epidemic Preparedness & Response, Beijing 100191, China; 6 Medical Research Council Population Health Research Unit at the University of Oxford, Oxford, United Kingdom

## To the editor

Chronic liver disease (CLD), including cirrhosis and liver cancer, accounts for 9.5 million deaths globally in 2017, one third of which occurred in China^1^. Non-alcoholic fatty liver disease (NAFLD) alongside its metabolic risk factors has become a major risk factor for CLD^2^. Based on easily accessible risk factors, the CLivD score, a simple model to predict future risk of CLD, has been developed and validated in European populations^3^. This model can be used to identify individuals at high risk who could benefit from lifestyle interventions and should be referred for further liver assessment. However, the associations of lifestyle and metabolic risk factors with CLD risk differ in Chinese adults^4,5^. It is unclear whether the CLivD score could be used in Chinese adults after model recalibration. Therefore, we performed an external validation of the CLivD score for Europeans in the China Kadoorie Biobank (CKB).

The China Kadoorie Biobank (CKB) recruited 512,715 participants aged 30-79 years from 10 areas (5 urban, 5 rural) in China during 2004-2008^6^. All participants completed an interviewer-administered laptop-based questionnaire and physical measurements were recorded by trained technicians. 17 biochemistry traits were measured in a subsample of 17,681 participants^7^. Follow-up for International Statistical Classification of Diseases and Related Health Problems 10th Revision-coded incident events continued to January 1, 2018, through linkage with morbidity and mortality registries and a nationwide health insurance system. CLD was defined as advanced liver disease or liver-related mortality, including liver cirrhosis, NAFLD, and liver fibrosis (ICD-10 code: K70, K72, and K74 alongside other complications of CLD)^3^. Discrimination was assessed using Harrell’s C index. Calibration was assessed graphically by comparing the predicted risks and the observed risk. We recalibrated the original CLivD score model by re-estimating the predictor coefficients in CKB (i.e. model refit)^8^.

Of the 504,009 participants included (excluding participants with a prior history of cancer, cirrhosis or hepatitis), the mean age was 52 years (SD 10.7 years), and 59.2% were women. During 11 years of follow-up, there were 4091 incident cases of CLD. The Harrell’s C index of the CLivD score was 0.65 (0.64-0.66) for model_non-lab_ and 0.68 (0.62-0.73) for model_lab_ (Figure). The Harrell’s C index for model_non-lab_ in CKB was similar to those reported by the original CLivD score paper, but the Harrell’s C index for model_lab_ in CKB was lower. For model_non-lab_, the Harrell’s C index was slightly lower among men and participants who tested positive for HBsAg and those with diabetes. For model_lab_, the Harrell’s C index was slightly higher among women and those from rural areas, weekly drinkers, and fatty liver index (FLI) ≥60 (Figure). Both models under-estimated risk at lower levels of observed risk but over-estimated risk among participants among the top decile of observed risk. After recalibration, both models still underestimated the risk (Figure).

Model_non-lab_ had good overall discrimination in this Chinese population. Three prospective studies have externally validated the CLivD score. The first two studies were European cohorts and reported a C-statistic of 0.65 (CCHS) and 0.74 (Whitehall II) for model_non-lab_, separately^6^. The third study was NHANES, involving diverse race and ethnicities, and showed a C-statistic of 0.637 for model_non-lab_^9^. The current study reported a C-statistic of 0.649 for model_non-lab_, which was generally consistent with the aforementioned studies. In contrast, model_lab_ performed worse in the Chinese population than previous studies. The C-statistic for model_lab_ was 0.78 in CCHS and 0.733 in NHANES, respectively, and was higher than the C-statistic of 0.68 in CKB. Possible reasons for the worse performance of model_lab_ included the much smaller number of participants with biochemistry data in CKB, their inclusion criteria and the weak associations between predictors and incident CLD.

The CLivD score under-estimated the risk of CLD in CKB across the range of observed risk even after recalibration by refitting the models. Underestimation was a particular issue in HBsAg-positive participants, which is anticipated since progression of liver disease is driven more by viral factors than the factors included in the CLivD score. In contrast, calibration was much better in the HBsAg-negative subjects. The unsatisfying performance of model calibration may reflect the fact that different sets of predictors are needed for Chinese to develop risk prediction models with good calibration as well as good discrimination.

This was the first study to externally validate risk prediction models for CLD in the CKB, a large, geographically diverse population-based cohort study in China. The model including easily obtainable variables based on demographic, lifestyle factors, and medical history was found to have good discrimination, whereas the model additionally including GGT had a lower discrimination. Both models underestimated the risk across the range of observed risk even after model refit. Future studies are warranted to develop risk prediction models for CLD in Chinese adults taking into account of the varying risk factors and aetiologies of CLD.

## Figures and Tables

**Figure:**
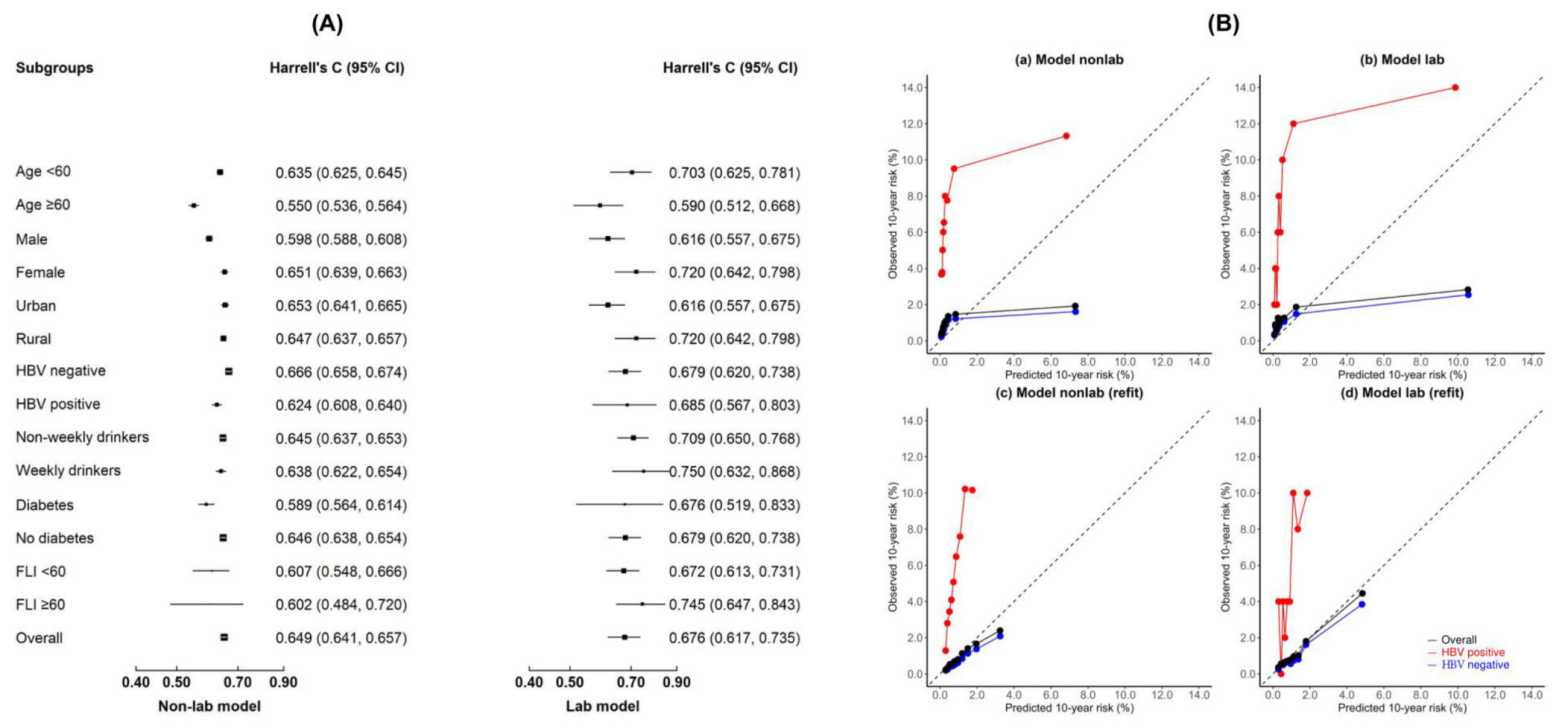
Harrell's C and model calibration curves for the CLivD score model in CKB Panel (A): Time since study entry was used as the underlying time scale. The squares represent C-statistics and the vertical lines represent 95% CI. The area of the squares is inversely proportional to the variance of the log C-statistic. Panel (B): Observed risk was plotted against expected risk of developing CLD over the 10-year period in groups based on quintiles of expected risk and the slope and intercept were estimated. (A) and (B) were for the original CLivD score models, while (C) and (D) were from model refit.
